# Single-stage minimally invasive resection of synchronous left atrial myxoma and pulmonary atypical carcinoid: case report

**DOI:** 10.1093/jscr/rjag540

**Published:** 2026-06-30

**Authors:** Feng Chen, Guozheng Gao, Shaobo Gao, Wei Zhang, Yijun Xu

**Affiliations:** Department of Thoracic Surgery, Tianjin Chest Hospital & Tianjin University Affiliated Chest Hospital, No. 261 Taierzhuang South Road, Jinnan District, Tianjin 300222, China; Department of Pathology, Tianjin Chest Hospital & Tianjin University Affiliated Chest Hospital, No. 261 Taierzhuang South Road, Jinnan District, Tianjin 300222, China; Department of Cardiac Surgery, Tianjin Chest Hospital & Tianjin University Affiliated Chest Hospital, No. 261 Taierzhuang South Road, Jinnan District, Tianjin 300222, China; Department of Cardiac Surgery, Tianjin Chest Hospital & Tianjin University Affiliated Chest Hospital, No. 261 Taierzhuang South Road, Jinnan District, Tianjin 300222, China; Department of Thoracic Surgery, Tianjin Chest Hospital & Tianjin University Affiliated Chest Hospital, No. 261 Taierzhuang South Road, Jinnan District, Tianjin 300222, China

**Keywords:** atrial myxoma, pulmonary atypical carcinoid, synchronous tumors, minimally invasive surgery

## Abstract

Synchronous primary cardiac and pulmonary tumors are rare, presenting complex therapeutic challenges regarding surgical sequencing and perioperative risk. A 52-year-old man with atrial fibrillation was incidentally diagnosed with a left atrial myxoma and a suspicious left upper lobe hilar mass. Following multidisciplinary evaluation, a single-stage minimally invasive approach was performed. The procedure involved a right mini-thoracotomy for atrial myxoma resection under cardiopulmonary bypass, followed immediately by video-assisted thoracoscopic left upper lobectomy with systematic lymph node dissection. Pathology confirmed atrial myxoma and stage IB (pT2aN0M0) pulmonary atypical carcinoid. The postoperative course was uneventful; the patient remains disease-free at 36 months. In selected patients, simultaneous minimally invasive cardiothoracic resection is feasible and effective. This integrated strategy addresses competing risks—such as systemic embolization and oncologic progression—in a single setting, supporting a risk-adapted alternative to traditional staged approaches.

## Introduction

Atrial myxomas are the most common benign cardiac tumors, yet they pose severe risks of systemic embolization and intracardiac obstruction, necessitating prompt resection. Conversely, pulmonary atypical carcinoids are neuroendocrine neoplasms requiring anatomical resection and systematic lymph node dissection due to their potential for nodal involvement and recurrence [1, 2].

The synchronous occurrence of these primary tumors is exceedingly rare, creating a complex therapeutic dilemma. Traditional staged strategies prioritize cardiac intervention to mitigate embolic risk but may delay oncologic treatment and increase cumulative perioperative stress. While simultaneous resections have been reported, they typically rely on high-morbidity open surgical techniques [3].

Video-assisted thoracoscopic surgery (VATS) has revolutionized thoracic oncology, offering outcomes comparable to open resection with reduced morbidity [4]. Integrating minimally invasive cardiac and pulmonary techniques into a single operative session may minimize surgical burden while ensuring cardiovascular stability and oncologic completeness.

We report the case of a 52-year-old male with paroxysmal atrial fibrillation presenting with a left atrial myxoma and a synchronous pulmonary atypical carcinoid. The patient was successfully managed via a single-stage, bilateral minimally invasive approach, demonstrating the feasibility of this integrated strategy for complex, dual-pathology cases.

## Case presentation

**Figure 1:**
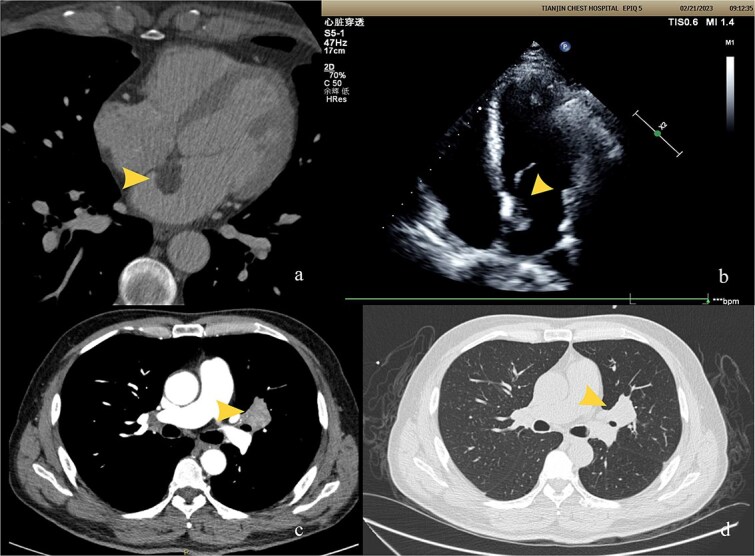
Preoperative imaging. (a) Contrast-enhanced CT showing a left atrial filling defect (arrow). (b) Transthoracic echocardiography demonstrating a pedunculated left atrial mass (arrow). (c, d) Contrast-enhanced CT revealing a left lower lobe hilar mass (arrows), suspicious for primary lung cancer.

A 52-year-old man presented with a 3-month history of paroxysmal palpitations. Holter monitoring confirmed paroxysmal atrial fibrillation (AF). Pre-ablation workup via contrast-enhanced computed tomography (CT) revealed a left upper lobe hilar mass and a left atrial filling defect. Transthoracic echocardiography identified a 2.0 × 1.3 cm pedunculated mass on the interatrial septum, consistent with a myxoma (Fig. 1).

A multidisciplinary team (MDT) prioritized a one-stage surgical strategy over staged procedures to minimize cumulative perioperative morbidity and prevent oncologic delay. The plan involved thoracoscopic resection of the atrial tumor under cardiopulmonary bypass (CPB), followed by VATS left upper lobectomy.

### Surgical procedure

Under general anesthesia with double-lumen intubation, the patient was placed in a 20° left lateral decubitus position. A right mini-thoracotomy with thoracoscopic assistance was performed. After femoral cannulation and CPB initiation, the left atrium was accessed under cardioplegic arrest. The pedunculated tumor was completely excised from the interatrial septum. Following de-airing and weaning from CPB with stable hemodynamics, the patient was repositioned for the thoracic phase.

A left-sided VATS approach was utilized for the lung resection. The left upper pulmonary vessels and bronchus were systematically divided using endoscopic staplers. Following intraoperative frozen-section confirmation of malignancy, a left upper lobectomy and systematic mediastinal lymph node dissection (stations 5, 6, 7, 10, and 11) were completed.

### Postoperative course and pathologic findings

**Figure 2:**
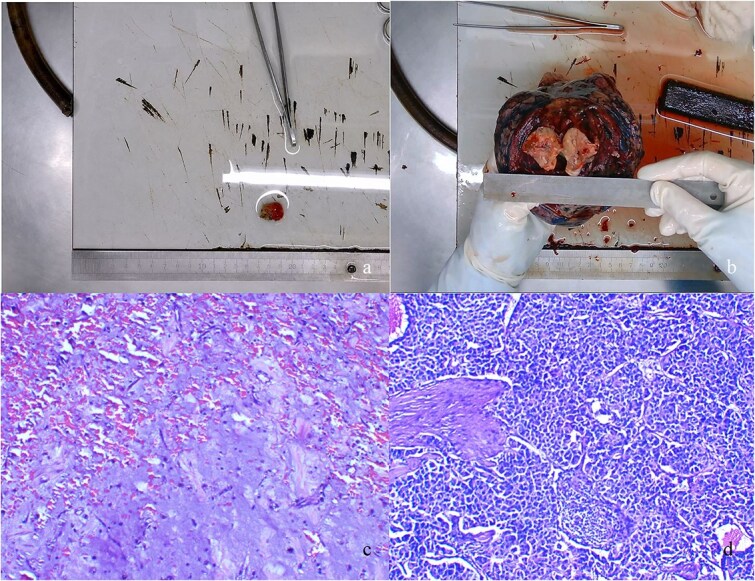
Pathologic findings. (a) Gross specimen of the resected left atrial myxoma. (b) Cross-sectional view of the resected left lower lobe tumor. (c) Hematoxylin and eosin (H&E) staining of the atrial myxoma (original magnification ×100). (d) H&E staining of the pulmonary atypical carcinoid tumor (original magnification ×100).

The postoperative course was unremarkable, except for transient AF managed with amiodarone. Histopathology confirmed a 2.5 cm left atrial myxoma. The pulmonary lesion was diagnosed as an atypical carcinoid (3.5 × 3.0 cm) involving the bronchial wall and lung parenchyma, with present intravascular tumor emboli. All resected lymph nodes were negative, resulting in a final pathologic stage of pT2aN0M0 (Stage IB) (Fig. 2).

**Figure 3:**
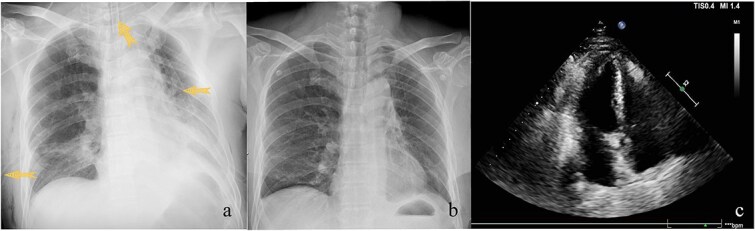
Postoperative imaging. (a) Chest radiograph obtained on postoperative day 1 demonstrating bilateral thoracic drainage tubes and an endotracheal tube (arrows). (b) Follow-up chest radiograph at discharge showing no evidence of pneumothorax or pleural effusion. (c) Postoperative transthoracic echocardiography confirming absence of residual left atrial mass.

The patient was discharged following satisfactory recovery (Fig. 3) and completed four cycles of adjuvant carboplatin and etoposide. At 36-month follow-up, he remains alive and disease-free.

## Discussion

The management of synchronous cardiac tumors and pulmonary malignancies is rare and lacks established guidelines, with evidence limited to isolated case reports [5, 6]. Interventions for these conditions are interdependent; the surgical strategy must balance the immediate embolic risk of a cardiac mass against the potential progression of a pulmonary malignancy.

Individualized assessment is essential for therapeutic sequencing. Prioritizing pulmonary surgery risks embolic or hemodynamic complications from the intracardiac mass, which can occur unpredictably even in asymptomatic patients [7]. Conversely, delaying oncologic resection risks interval progression and compromised resectability. A simultaneous approach was selected here to mitigate embolic risk, prevent oncologic delay, and reduce the cumulative physiological burden of staged procedures. Critically, observational data suggest that CPB does not adversely impact long-term cancer-specific survival in patients with solid tumors, supporting the oncologic safety of integrated procedures [8].

Advances in minimally invasive techniques further enhance the feasibility of simultaneous resection. Right mini-thoracotomy for atrial myxoma achieves excision completeness and recurrence rates comparable to median sternotomy while accelerating recovery [9]. Similarly, VATS lobectomy provides long-term survival equivalent to open thoracotomy for early stage lung cancer with significantly reduced morbidity [4]. These combined modalities allow for complex multi-organ surgery without the substantial operative trauma of traditional open approaches.

The operative sequence—cardiac resection followed by pulmonary lobectomy—was structured to eliminate thromboembolic risk before thoracic manipulation. Ensuring hemodynamic stability after weaning from CPB was a prerequisite for progressing to the pulmonary phase. Maintaining oncologic rigor was paramount; we performed a VATS lobectomy with systematic lymph node dissection, which remains a critical determinant of staging accuracy and prognosis in pulmonary neuroendocrine tumors [10].

The presence of intravascular tumor emboli in the final pathology of this atypical carcinoid underscores the necessity of timely resection to prevent systemic spread. While staged management has been the traditional default, this case demonstrates that a simultaneous minimally invasive approach is a feasible and effective alternative. It allows for definitive treatment of dual pathologies in a single setting, provided there is careful patient selection and multidisciplinary coordination.

In conclusion, synchronous cardiac and pulmonary tumors require risk-adapted planning. A single-stage minimally invasive strategy can achieve oncologic completeness while preserving cardiovascular stability and minimizing perioperative morbidity.

## Data Availability

Clinical data supporting this case report are held by Tianjin Chest Hospital and are available upon reasonable request to the corresponding author (Yijun Xu) with ethical committee approval, due to patient confidentiality.
